# Characterization of the complete chloroplast genome of *Caulerpa cupressoides* (Bryopsidales, Chlorophyta)

**DOI:** 10.1080/23802359.2018.1501294

**Published:** 2018-08-13

**Authors:** Hong Yan, Yuan Yuan, Qiang Qiu, Dahai Gao

**Affiliations:** a Center for Ecological and Environmental Sciences, Northwestern Polytechnical University, Xi’an, China;; b Qingdao Research Institute, Northwestern Polytechnical University, Qingdao, China;; c Department of Marine Organism Taxonomy and Phylogeny, Institute of Oceanology, Chinese Academy of Sciences, Qingdao, China

**Keywords:** *Caulerpa cupressoides*, chloroplast genome, Bryopsidales

## Abstract

*Caulerpa cupressoides* (Vahl) C. Agardh is a widely distributed tropical green algae. The circular chloroplast genome was 130,895 bp in length, with a GC content of 34%. In total, 99 genes were identified and they were consisted of 63 coding genes, 30 tRNA genes, and 6 rRNA genes. This chloroplast genome did not show an obvious quadripartite structure. Phylogenetic analysis revealed that *C. cupressoides*, *C. racemosa*, and *Tydemania expeditionis* were close relatives, with high bootstrap values. The characterized complete chloroplast genome of *C. cupressoides* will provide essential date for further studies of Bryopsidales.

*Caulerpa cupressoides* (Vahl) C. Agardh is a siphonous green seaweed belonging to the genus *Caulerpa* and distributed in tropical seas (Nielsen and Price 2001). Recently, the antinociceptive and anti-inflammatory activities of the lectin isolated from this species have been proved (Benevides et al. 2001; Vanderlei et al. 2010; Da et al. 2014), indicating its potential medical value. In this study, we reported a complete cp genome of the *C. cupressoides*.

The fresh thallus of *C. cupressoides* was collected from Sanya (Hainan, China; 109°30′11.52″E, 18°12′36.72″N), and were used for the total genomic DNA extraction with the modified CTAB method (Doyle 1987). Voucher herbarium specimens were prepared and deposited in Marine Biological Museum of the Chinese Academy of Sciences (MBMCAS). The whole-genome sequencing was conducted with 150 bp pair-end reads on the Illumina Hiseq Platform (Illumina, San Diego, CA). In total, 2.3 million high quality base pairs of sequence data were obtained and the genome was assembled with NOVOPlasty (Dierckxsens et al. 2017). Protein-coding genes and non-coding RNAs were annotated with GeSeq (Tillich et al. 2017). The annotations chloroplast genome was submitted to GenBank database under Accession (No. MG797569). The phylogenetic relationship of *C. cupressoides* was conducted based on the complete chloroplast genome of 14 species in Trebouxiophyceae and Ulvophyceae. The complete cp genome sequences were aligned by MAFFT software (Katoh et al. 2002) and the phylogenetic tree was constructed using RAxML (Stamatakis 2014) by maximum-likelihood method.

The complete chloroplast genome of *C. cupressoides* was 130,895 bp in length. The chloroplast genome contained 99 genes, including 63 protein-coding genes, 30 transfer genes, and 6 ribosomal RNA genes. Like congeneric *C. racemosa*, this chloroplast genome did not show a quadripartite structure and lacked the large rRNA operon-encoding Inverted Repeat (IR). The number of tRNA and rRNA were same between *C. cupressoides* and *C. racemose*, while the number of genes was 7 less than *C. racemose* due to few ORFs genes. The overall base composition was 32.2% for A, 17.4% for C, 16.6% for G and 33.8% for T.

The phylogenetic tree analysis showed that *C. cupressoides* was clustered with *T. expeditionis* and *C. racemose* (Figure 1). However, the species *C. cliftonii* of the same genus was not similar. Our results revealed that it was quite similar to previously published trees (Lam and Zechman 2006; Fučíková et al. 2014; Leliaert and Lopez-Bautista 2015; Melton III et al. 2015). To date, the classification of Trebouxiophyceae and Ulvophyceae was not clear enough compared to previous studies. The chloroplast genome traits have the potential to provide insights of deep-level relationships at the Bryopsidales level.

**Figure 1.**
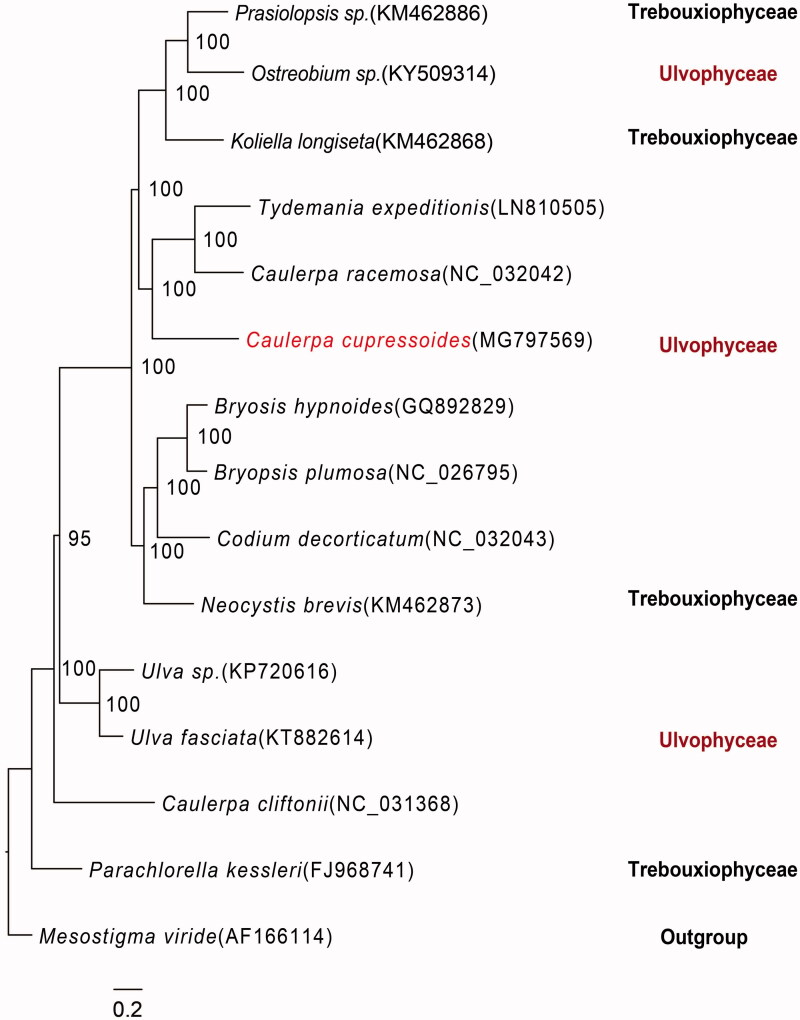
Phylogenetic tree of 15 species based on chloroplast genome. *Mesostigma viride* was used as an outgroup. Sp. represents an uncertain species.
